# Microscale Computed Tomography (μCT) Imaging of Leak Pathways for Optimized Leak-Free 3D Printed Fluidics

**DOI:** 10.1021/acsapm.5c02274

**Published:** 2025-10-24

**Authors:** Rowan Leeder, Kathryn E. Rankin, Adrian M. Nightingale

**Affiliations:** † Mechanical Engineering, Faculty of Engineering and Physical Sciences, 7423 University of Southampton, Southampton SO17 1BJ, U.K.; ‡ μ-VIS X-ray Imaging Centre, Faculty of Engineering and Physical Sciences, University of Southampton, Southampton SO17 1BJ, U.K.

**Keywords:** 3D printing, computed tomography, fused filament fabrication, fused deposition modeling, polypropylene−ethylene copolymer, microfluidic, millifluidic

## Abstract

3D printing is a highly attractive method for manufacturing micro- and millifluidic devices due to fast fabrication times and a low barrier to entry. Of the common 3D printing methods, fused filament fabrication (FFF) is the most accessible but is also susceptible to leakages when using default printer settings. Here, we combine microscale computed tomography (μCT) X-ray imaging with bulk leak testing to understand the fundamental structural reasons why leakages occur, and the effect of optimizing print parameters. In contrast to previous recommendations, we show that the amount of infill can be reduced as required, with print bodies being intrinsically porous, regardless of infill. Instead, we find that it is solely the channel wall quality that determines whether leaks will occur. In keeping with previous reports, we see that smaller layer heights (<0.1 mm) and increased flow rates (>100% compared to the recommended rate) are key to preventing leakage, and show this is because of their positive effect on channel wall formation. A key consequence of being able to maintain channel integrity while using low infill values is that print times and material costs can be greatly reduced (over 50% time and cost savings for the test pieces used here) without compromising device performance.

## Graphical abstract

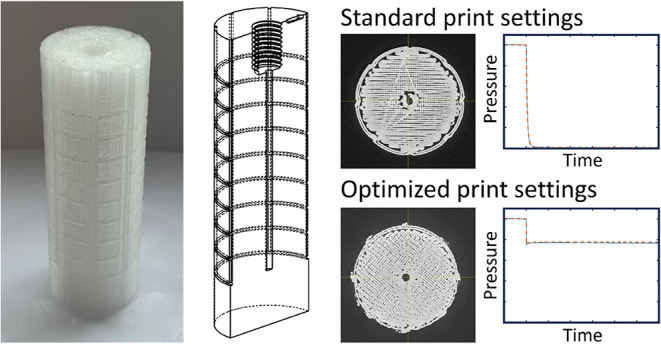

## Introduction

3D printing is an increasingly popular tool for fabricating micro- and millifluidic systems.^1,2^ While they cannot reach the submicrometer resolution of devices replica-molded from lithographically fabricated masters, they offer advantages in terms of speed, cost, and accessibility. Fused filament fabrication (FFF, also often referred to as fused deposition modeling, FDM) and photocure printing (e.g., stereolithographic addition, SLA) are the most common methods due to the wide availability of low-cost printers. In FFF printing, thermoplastics are extruded through a heated nozzle that can move in the *x*–*y* plane, such that the nozzle puts down a series of pathways to build up a two-dimensional pattern. If the pattern is on a movable *z*-stage, two-dimensional layers can be built up, one upon the other, to generate three-dimensional features. While the spatial resolution on FFF printing is slightly inferior to photocure methods,^3^ prints can be made in a much wider range of materials, with different mechanical and electrical properties, and excellent chemical compatibilities. Furthermore, external items (e.g., electrodes, optics, membranes) can be more easily incorporated to expand the functionality of finished printed devices.^4–6^ FFF printing has allowed users to design and fabricate a range of bespoke chemical processing technology, including flow reactors with 3D mixing elements,^7^ photochemical reactors,^8^ redox flow batteries,^9^ chromatography columns,^10^ and filtration and separation devices.^6^

A challenge with FFF-printed fluidics, however, is that they typically leak when fabricated using default print settings. Users can control a range of print setting options to tailor the print and, for leak-free fluidics, recent papers have recommended using low layer heights (0.1 mm or less^3,11–16^ ), increased flow rates (typically 4–10% greater than the software-recommended flow rate^8,12–15,17^ ), and 100% infill^3,8,11–14,16,17^ (where infill determines how much material is deposited in the print interior). The recommended print settings have been arrived at empirically, but are hypothesized to prevent leakages by removing small air gaps between neighboring pathways^13^ that can result from the rounded pathway cross sections.^18,19^ In this work, we use microfocus X-ray computed tomography (μCT) X-ray imaging to find evidence for the leak pathways—linking macroscopic observations of leakage with microscopic observations of the internal structure of printed parts and hence arriving at an informed understanding of how to optimize print parameters for printing fluidics.

## Experimental Section

Standardized test pieces were designed with a single channel (1.5 mm diameter, 40 mm length), which was closed at one end and had a female 1/4–28" fitting at the other end to connect to external tubing. The channel width was deliberately chosen to be conservatively wide and easily printable to ensure reliable printing. Two variants of this design were implemented to allow the main channel to be positioned vertically (Figure 1a–c) or horizontally (Figure 1d–f) while ensuring that the 1/4–28" fitting was always printed in a vertical orientation—ensuring the screw threads printed well and that the sealing surface (at the bottom of the fitting) was flat and smooth to allow reliable sealing to a flangeless fitting (IDEX). In each model, the channels were surrounded by 9.7 mm of solid material, and markings were patterned into the outer surface so that the position of any external leaks could be related back to the print orientation.

**Figure 1:**
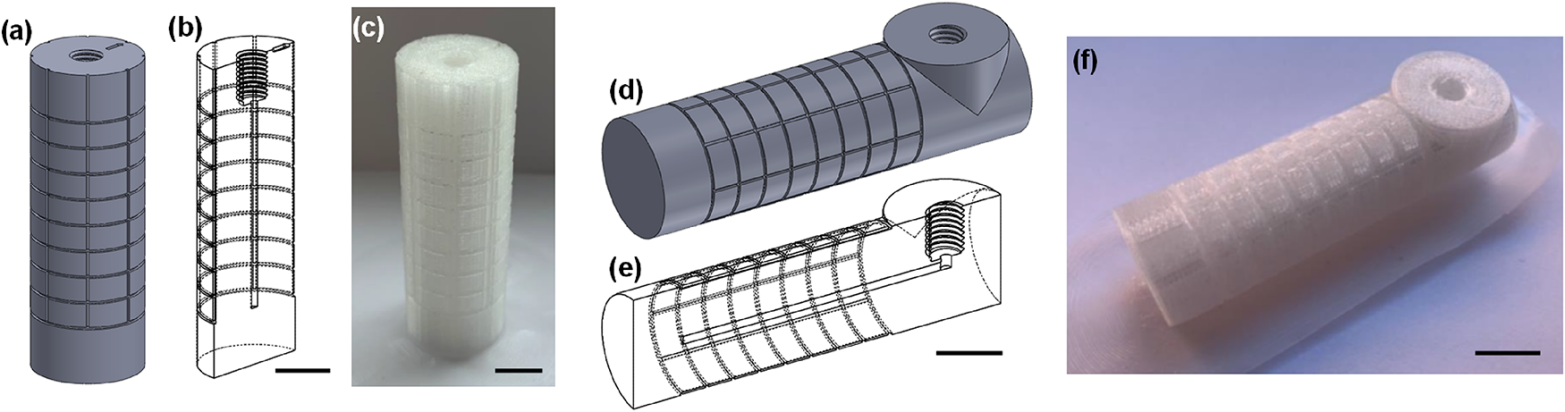
Images showing the test pieces printed with vertical channels (a–c) and horizontal channels (d–f). In each case, the design is shown as a solid device (a, d), in cross section (b, e), and as printed (c, f). All scale bars are 1 cm.

All test pieces were printed on an UltiMaker 3 printer fitted with a 0.4 mm nozzle using an UltiMaker-brand polypropylene filament. Like most commercial polypropylene filaments, consultation of the safety data sheet shows that the material was in fact a polypropylene–ethylene copolymer. Polypropylene was used, as this is the most chemically compatible of the commonly available filament materials and hence best suited to fluidic applications. It has been widely used in studies of FFF-printed fluidics^6,12–14,18^ and more generally in 3D printed reactors.^5,8,16,17,20–22^ Pieces were designed in SolidWorks, exported as .stl files, and then imported into UltiMaker Cura to prepare print settings. The default settings for polypropylene were used, with the exception of layer height, flow rate, and infill, which were adjusted as later described. All pieces were printed individually in the center of the build plate. UltiMaker-brand adhesion sheets were used on the build plate to ensure good contact between the build plate and the first layer of each print.

Leak testing was done in two ways. For quick screening, a manual approach was used, whereby a 10 mL disposable syringe (BD Plastipak) was connected to the test piece and pressure applied by hand, giving gauge pressures >160 kPa. After screening, a more quantitative approach was used, which involved exposing the test piece to an elevated pressure and then tracking what happened to the system pressure over time: each test piece was connected in series to (a) an open/close manual valve (Idex P-782), (b) a pressure sensor (NXP MPX4250A, connected using a T-junction, Idex P 713) used to quantify the system pressure, and (c) a syringe pump (KD Scientific KDS 100, using a 10 mL BD Plastipak syringe) used to pressurize the system. Throughout, 1/4–28" flangeless fittings (IDEX) and 2 mm inner diameter PTFE tubing were used. The pressure sensor was connected to an Arduino Nano microcontroller, which in turn fed readings to a desktop computer running a LabVIEW script (developed in-house) to continually record the system pressure. During testing, the valve was initially closed, and the syringe pump ran (1 mL/min) until the gauge pressure increased to approximately 150 kPa. When the required pressure had been achieved, the syringe pump was stopped, and the pressure reading was left to stabilize (∼30 s). The valve was then opened to expose the test piece to the pressurized side of the system, and the ensuing pressure trend was recorded.

For μCT imaging, test pieces were scanned using a Nikon XTEK XTH 225 kVp microfocus CT system with a PerkinElmer XRD 1621 CN14 HS detector (PerkinElmer Optoelectronics, Germany) and Tungsten target material. The X-ray conditions were set as 100 kVp peak voltage and 238 μA current, and the source to object and source to detector distances were set as 100 and 798 mm, respectively. Using an exposure time of 250 ms and 24 dB analogue gain on the detector, 1501 projection images were acquired throughout 360° rotation of the test piece, using the minimize ring artifacts acquisition mode and averaging 4 frames per projection.

Projection data were reconstructed into 32-bit float volumetric data sets (1000 × 1000 × 2000 voxels) using the filtered back-projection algorithms implemented within CTPro3D and CTAgent software v6.2 (Nikon Metrology, U.K.). The resulting voxel resolution was 25 μm. Each 32-bit raw volume was downsampled to 8-bit using ImageJ/Fiji (Rasband, W.S., ImageJ, U.S. National Institutes of Health, Bethesda, Maryland, https://imagej.nih.gov/ij/, 1997–2019) to reduce data processing time.

ImageJ/Fiji was used to compare the volume fraction of porosity within the theoretically solid wall surrounding the central channel. The channel of each test piece was aligned vertically with the *Z* axis (by reslicing the volume in *XZ* and *YZ* and using Image → Rotate), and a 59 voxel diameter circular region (1.5 mm diameter) was specified at the channel to indicate the channel region as in the test piece CAD model. A 120 voxel diameter circular region with coordinates centered with that of the channel region was then specified to indicate the 3 mm outer diameter of the nominally solid wall surrounding the channel. The volume was cropped, and the slice range in *Z* was set as the nominal height of the channel (120 × 120 × 1595 voxels, 3 × 3 × 40 mm height). A global thresholding method (Otsu) was used to segment the volume into regions corresponding to air/porosity (0–140) and material (141–255) using a black background of binary masks. By analyzing the histogram stack, the count of voxels corresponding to air (0) and material (255) within the nominally solid wall region was used to calculate the void volume fraction for comparison (voids in wall volume/total wall volume).

## Results

Previous literature reports of leak-free fluidics recommend using 100% infill, low layer heights (0.1 mm or lower), and increased flow rates.^3,8,11–15^ Of these parameters, the infill makes the most intuitive sense, as it should leave no spaces in the bulk of the print for fluid to leak into. Hence, we began by keeping infill constant at 100% and investigating the role of overextrusion (increased flow rate) and layer height. Overextrusion is the most notable of these parameters as it is not normally used for standard (nonfluidic) printing applications, and the option to control this parameter is not easily accessible within slicing software, in contrast to layer height or infill. By increasing the flow of the plastic without increasing the distance between layer paths or layer height, the width of the extruded pathway (i.e., the width of the molten plastic trail put down by the moving nozzle) increases, such that it should better contact (and hence better bond with) neighboring pathways in each 2D print layer. An inherent disadvantage of overextrusion, however, is that it will drive printed dimensions away from their nominal sizes (increasing dimensions of positive features in the *x*–*y* plane, decreasing dimensions of negative features), increasing the need for empirical dimension optimization, and hence, overextrusion should be avoided if possible.

Testing began with the test pieces with vertical channels (Figure 1a–c). Multiple versions were printed, with layer heights ranging between 0.06 and 0.2 mm (the standard range suggested by the slicing software for this printer and material) and extrusion rates of 95, 100, and 110%. Figure 2a shows the results of manual leak testing. At the lowest layer height, no leaks were detected in any of the test pieces, even when the plastic was underextruded at 95%. Extrusion rate also had a positive effect, most notably at the higher layer heights, for example, the 0.15 mm layer height print was only leak-free at 110% extrusion. To ensure reproducibility, test pieces at a range of layer heights (0.06, 0.1, and 0.2 mm, with 100% extrusion) and extrusion rates (95, 100, and 110%, with 0.1 mm layer height) were reprinted and tested again. All repeat test results reproduced the original findings. For all pieces that leaked, the observed external position of the leaks varied randomly and could not be linked to print orientation.

**Figure 2:**
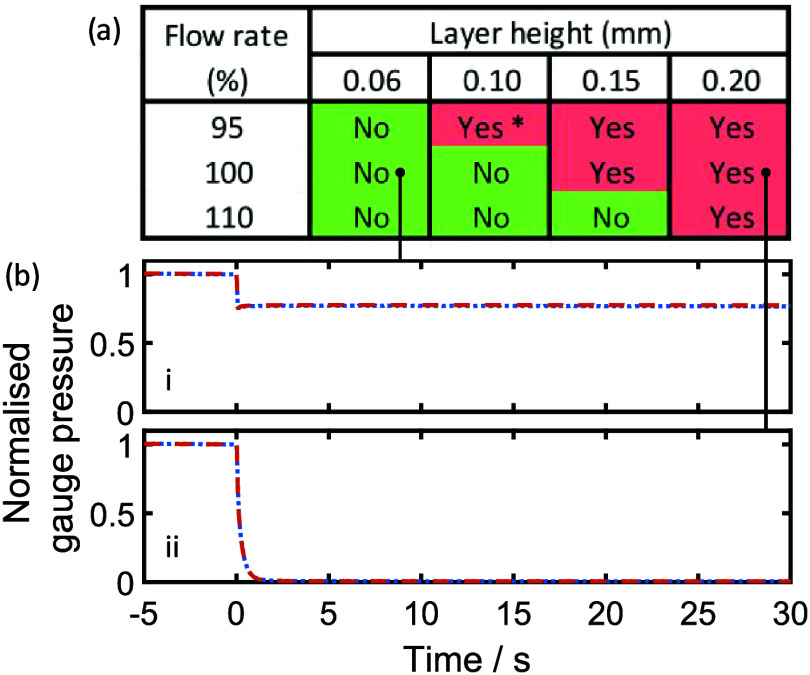
(a) Manual test results for test pieces with a vertical main channel, printed with varying layer heights and flow rates, showing whether leaking was observed. For the 0.10 mm layer height, 95% flow rate sample (result marked “*”), leaking was only observed at elevated pressures. Example results for two quantitative tests are shown below: (b­(i)), a leak-free test piece printed with 100% flow rate, 0.06 mm layer height, and (b­(ii)), a leaking test piece printed with 100% flow rate, 0.20 mm layer height. In both cases, the valve exposing the test piece to the elevated pressure was opened at *t* = 0. For both quantitative tests, two separate measurements of the same device are shown (red dashed and blue dotted lines). In each case, the lines overlay each other, showing the measurements to be repeatable.

The manual results were subsequently checked with quantitative testing in which test pieces were exposed to a prepressurized fluidic manifold and the drop in pressure monitored. All quantitative tests were consistent with manual testing, with representative results shown in Figure 2b. When a leak-free piece was tested (Figure 2bi), the pressure drop on exposure was finite, consistent with the pressure dissipating across an increased volume but immediately stabilizing due to the absence of leaks. By contrast, when a leaky test piece was exposed to the pressurized system (Figure 2bii), the pressure continuously dropped until the system was completely depressurized, consistent with fluid being freely lost. Repeats of both tests with a second set of test pieces reproduced the results (Figures S1 and S2). These findings correlate well with previous reports that emphasize the positive effect of overextrusion (high flow rates) and low layer height; however, it is notable that test pieces printed with the lowest layer heights (≤0.1 mm) did not require overextrusion. The avoidance of overextrusion, where possible, would allow printed dimensions to more closely match the nominal dimensions as defined in the original design.

We then imaged identical untested test pieces to investigate the structural causes of the observed leaks. Figure 3 shows reconstructed μCT slice images of test pieces printed with differing layer heights (a–d) but the same extrusion flow rate (100%), shown as vertical (i) and horizontal (ii–v) cross sections. The horizontal cross sections (on a parallel plane to the print bed) clearly show the two-dimensional printing pathways taken by the printhead as it lays down each layer. It shows the exterior and internal fluidic channels clearly defined by “wall” pathways with infill, put down as parallel lines, in between. Despite the nominal 100% infill setting, the body of each print is far from being a solid monolithic piece. Air gaps are visible within the interior of all test pieces, irrespective of the layer height setting. The porousness of the test pieces indicates that the main print body will have minimal to no effect on preventing leakage, and hence, it is the integrity of the walls, and in particular the walls of the fluidic channel, that will determine whether a piece leaks or not.

**Figure 3:**
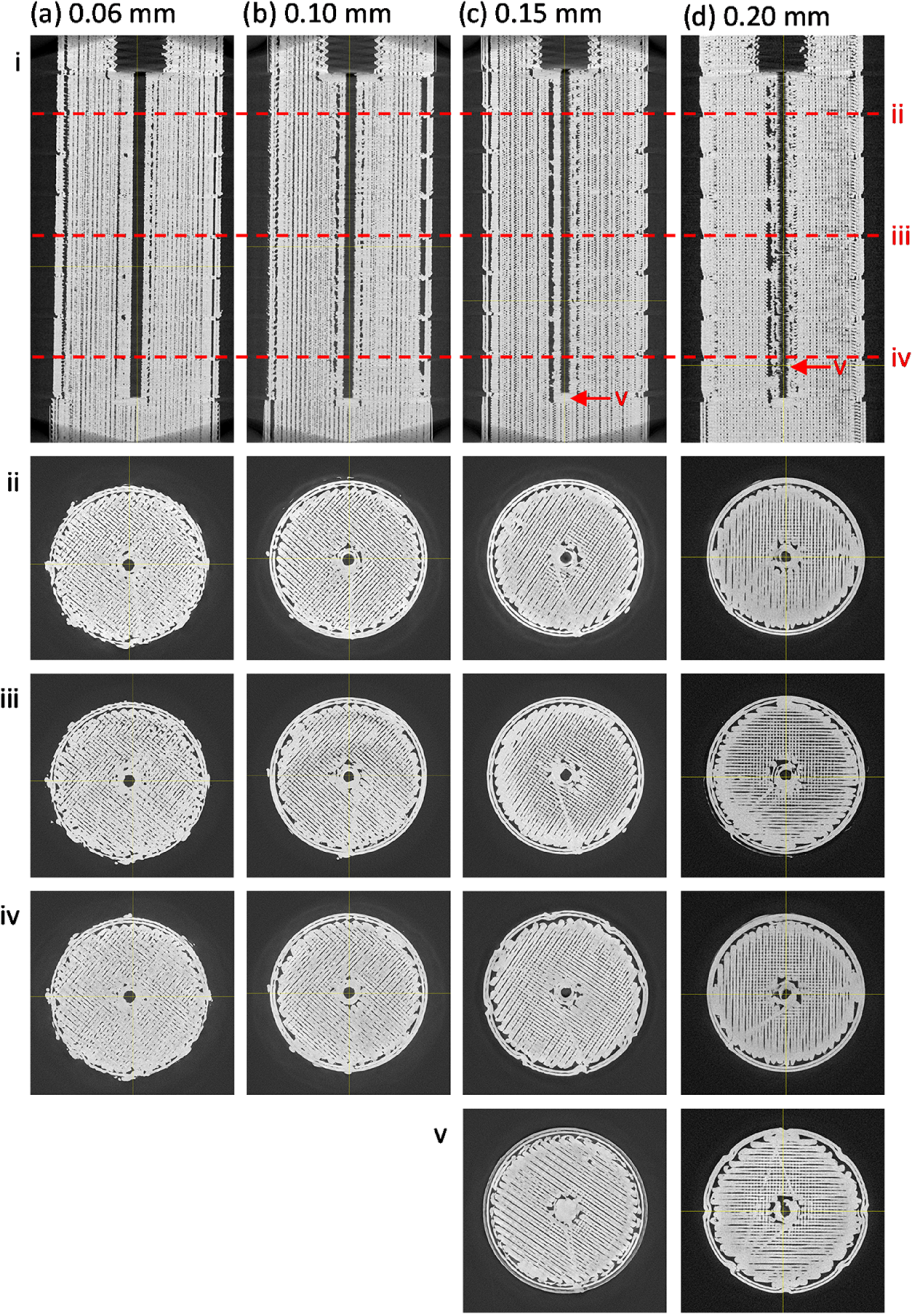
Reconstructed μCT slice images of test pieces with a vertical channel printed at 100% flow rate and layer heights of (a) 0.06 mm, (b) 0.10 mm, (c) 0.15 mm, and (d) 0.20 mm. Each is shown in vertical profile (i) and below in horizontal cross section (ii, iii, and iv) at the positions indicated by the red dotted lines in (i). Additional cross sections (v), marked by red arrows in (I), show the intact base of the channel for the 0.15 mm test piece (c) and a very clear break in the channel wall for the 0.20 mm test piece (d).

The print quality of the channel walls correlated well with the corresponding manual leak testing: At low layer heights (e.g., 0.06 mm layer height, Figure 3a), the cross sections show the channel walls to be consistently well printed, with no visible gaps and a good connection between walls and infill. As the layer height increases (Figure 3b–d), the quality of the channel walls visibly deteriorates. At the extreme of 0.2 mm, gaps are clearly visible (Figure 3dii–iv) with clear pathways from the channel into the infill area (Figure 3a­(v)). These qualitative observations match well with the quantification of the void fraction within the channel wall (Figure 4), which increases from 5.2% at 0.06 mm layer height to 14.0% at 0.20 mm layer height. While void fraction is not a direct measure of leak pathways, as it gives no information on the connections across the channel, we would expect a greater chance of leak pathways forming as the void fraction increases.

**Figure 4:**
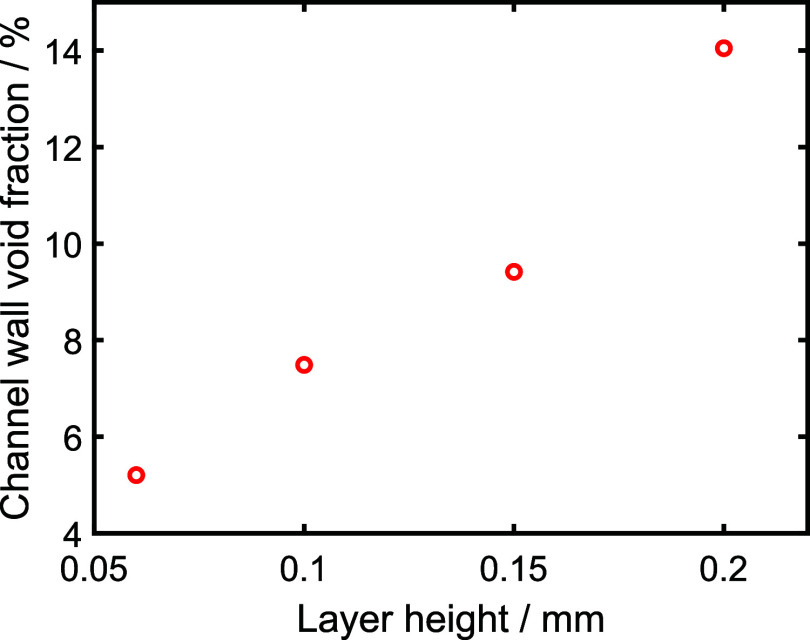
Void fraction within the channel wall shown relative to the layer height setting for test pieces printed with vertical channels and 100% flow rate.

The high quality of the low-layer-height prints is consistent with the bulk leak testing (where overextrusion was not required) and shows how leak prevention is consistent with the quality of the channel walls. This correlates well with a previous report that found increasing wall size (i.e., the number of wall pathways used to define each feature) had a positive effect on leak prevention,^23^ though in our own testing, we found wall size had no impact on leakage (data not shown).

The importance of layer height here is likely due to the vertical orientation of the channels during printing, where the connection between layers (rather than between pathways in each layer) is of optimum importance. Small layer heights will generate a pathway cross section with a higher aspect ratio,^18,24^ which will lead to an increased contact area between layers.

To ascertain whether the findings for the vertical channel test pieces were more generally applicable to other channel orientations, we then examined test pieces printed with horizontal channels (Figure 1d–f). As was the case for the vertical channel test pieces, the test pieces with horizontal channels were printed with different layer heights and flow rates, while keeping the infill constant at 100%.

As before, leaks could be clearly identified from manual testing (Figure 5a), and this was corroborated by quantitative testing (Figure 5b). Again, the positions of all external leaks varied randomly and could not be linked to print orientation. The relative importance of the different print parameters was notably different compared to the vertical channel test pieces, however. Here, flow rate was the most important parameter, with overextrusion a requirement for leak-free test pieces, irrespective of layer height (Figure 5a). The reason behind this can be seen by looking at the interior structure. Figure 6 shows three test pieces printed with differing print parameters, where (a) and (c) both leaked and (b) was leak-free. In each case, the internal structure is shown as vertical cross sections along the long (i) and short (ii) dimensions, with horizontal cross sections (on a parallel plane to the print bed) additionally shown for one test piece (Figure 6a­(iii–v)). Again, the bulk interior of all test piece bodies is seen to be porous (though this was less pronounced when overextruding at 110% flow rate, Figure 6b). The internal porosity again shows that if prints are to be leak-free, fluid must be contained by the channel walls, and hence these must be printed without gaps or breakages. Figure 6a­(iii–v) shows how the channel walls were constructed. Across the middle of the channel (Figure 6a­(iv)), filament has been put down around the perimeter of the channels (similar to the cross section of the vertical channels shown in Figure 3); however, the top Figure 6a­(iii) and bottom (Figure 6a­(v)) of the channels are capped with 2D flat plates, constructed by putting down filament in a zigzag pattern, similar to that used to infill the bulk of the test pieces. It is in those two-dimensional top and bottom pieces that imperfections in the channel wall are visible in the leaky test pieces (Figure 6a­(ii,iii,v) and c­(ii)). A good seal between neighboring pathways in the 2D print plane is therefore key to having a watertight seal and explains why overextrusion is more important for these horizontal channels than for the test pieces with vertical channels; overextrusion increases the width of the bead (while maintaining the same height), increases the contact between neighboring paths, and hence reduces chances of gaps in the plate structures that cap the top and bottom of the channels.

**Figure 5:**
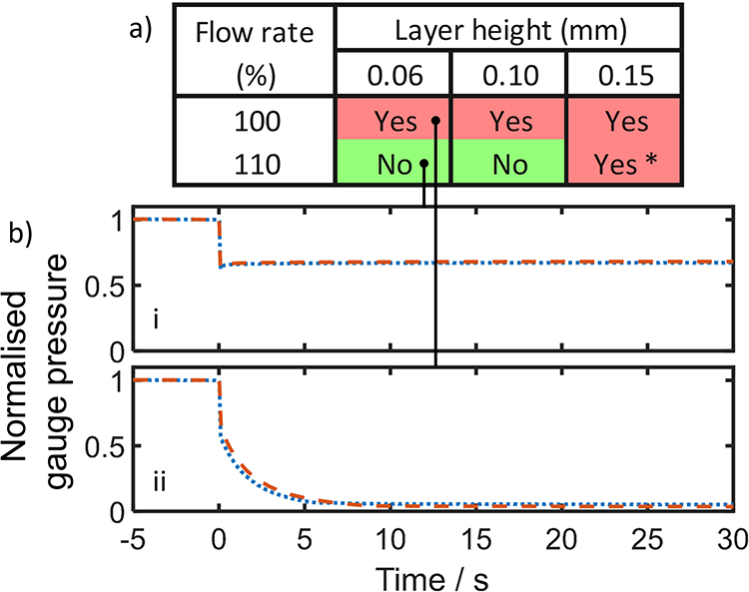
(a) Manual test results for test pieces with a horizontal main channel, printed with varying layer heights and flow rates, showing whether leaking was observed. For the 0.15 mm layer height, 110% flow rate sample (marked “*”), leaking was only observed at elevated pressures. The results of two quantitative tests are shown below: (b­(I)), a leak-free test piece printed with 110% flow rate, 0.06 mm layer height, and (b­(ii)), a leaking test piece printed with 100% flow rate, 0.06 mm layer height, where *t* = 0 represents the moment the pressurized system was exposed to the test pieces. For both quantitative tests, two separate measurements of the same device are shown (red dashed and blue dotted lines), and in each case, the lines overlay each other, showing the measurements to be repeatable.

**Figure 6:**
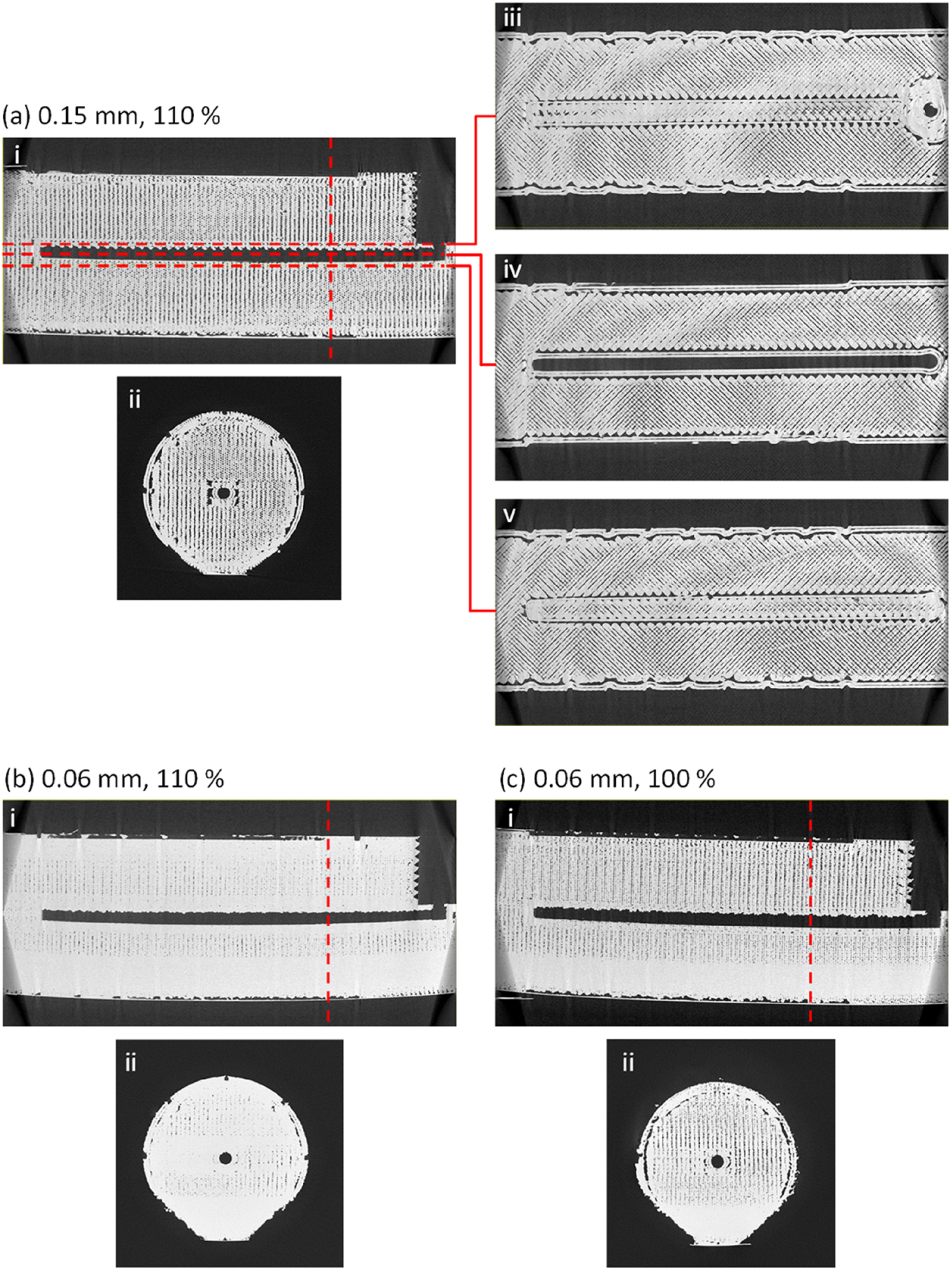
Reconstructed μCT slice images of test pieces with a horizontal channel printed with (a) 0.15 mm layer height and 100% flow rate, (b) 0.06 mm layer height and 110% flow rate, and (c) 0.06 mm layer height and 100% flow rate. For (a), the internal structure of the piece is shown as a vertical cross section along the length of the piece (i), a vertical cross section across the width (ii), and three horizontal cross sections positioned immediately above (iii), at the same height as (iv), and immediately below (v) the channel. The dashed red lines in (i) correspond to the position of the cross sections in (ii)–(v). For parts (b) and (c), the internal structure is shown via a vertical cross section along the length of the piece (i) and a vertical cross section across the width (ii). The dashed red lines in each (i) correspond to the position of the cross section shown in (ii).

Interestingly, the porousness of the test piece bodies and the importance of channel wall integrity suggest that the amount of infill should make no difference to whether a piece leaks or not. To test this, we printed a vertical channel test piece with 20% infill, a layer height of 0.06 mm, and 110% flow rate (Figure 7). The resulting test piece had well-defined contiguous channel walls (Figure 7a,b) and was consequently leak-free (Figures 7c and S3). The void fraction within the channel walls was determined to be 6.5%, consistent with previous measurements of leak-free devices (see Figures 2 and 4). The ability to reduce infill without compromising leak integrity is significant, as reducing the infill reduces print time (e.g., 4.75 h vs 10.75 h for the vertical test piece here) and material use (9 vs 20 g here).

**Figure 7:**
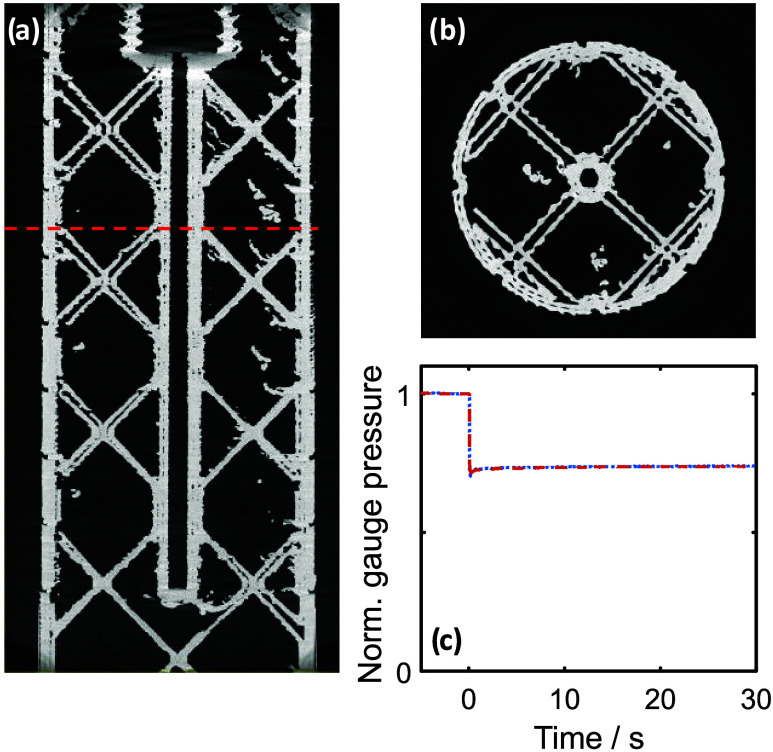
(a, b) Reconstructed μCT slice images of test pieces with a vertical channel printed with 0.06 mm layer height and 100% flow rate, shown as a vertical horizontal cross section (a), with a red dashed line showing the location of a corresponding horizontal cross section (b). Quantitative testing results of the same test piece are shown in part (c). Two separate measurements of the same device are shown (red dashed and blue dotted lines), which overlay each other, showing the measurements to be repeatable.

While the findings described here, using poly­(propylene–ethylene) copolymer, are expected to be broadly applicable to most common FFF filament materials and printers, we anticipate variations with the material and printer. Leakage prevention is dependent on forming good bonds between extruded plastic paths, and this is determined by the rheological properties of the plastic and the temperature during the deposition process, which in turn is related to the printer and print settings.^25^

It has previously been observed that different materials will have different behavior on leaving the nozzle (e.g., solidification rates), which affects bonding.^26–28^ Preliminary tests carried out in our lab have shown that the same qualitative trends that we report here are seen when using other common filament materials (i.e., lowering layer heights and overextruding prevents leakage), which we intend to explore in more detail in a later publication.

Cooling rates will be dependent on the printer and the printer settings;^26,29^ hence, it is reasonable to expect that different printers might require different print settings. In particular, we note that the printer used here was open on two sides; hence, we might expect different ambient temperatures and cooling rates when compared to printers that are completely open, or completely contained and temperature-controlled. Similarly, differences might be seen depending on where the print was located on the build plate and how this affects the ambient temperature.^29^

Finally, we note that we have focused on preventing leakage by ensuring that the channel walls are intact. A contrasting approach might be to instead focus on reducing the porosity of the bulk. This could be done, for example, by replacing the standard infill pattern with injection printing,^30^ a technique whereby walls are first printed and then large volumes of material extruded into the interior space to create a monolithic void-free interior. While this could be a viable approach, it is not a standard print option for slicing software and requires bespoke coding, making it inaccessible to most users. Moreover, it does not offer the advantages of faster print times and lower materials cost that are possible with well-formed channel walls and low infill (Figure 7).

## Conclusion

In summary, these results confirm previous reports that low layer heights and increased flow rates lead to leak-free devices, but show that these print parameters are of different importance depending on whether the channels are vertical or horizontal. Moreover, μCT scans show that the underlying reason for these parameter choices is to ensure that channel walls are well-formed. For vertical channels, low layer height (≤0.1 mm) is most important to ensure channel wall integrity, while overextrusion (>100%) is the determining factor for horizontal channels. Hence, both are recommended for most prints where channels could be in a range of orientations. The importance of well-formed channel walls also means that, when using optimized layer height and flow rate settings, infills can be set much lower to greatly decrease print times and material usage—in contrast to previous literature recommendations of 100% infill.

## Supplementary Material



## Notes

Additional quantitative leak testing results (PDF)
