# Supplementary material for: Microscale Computed Tomography (μCT) Imaging of Leak Pathways for Optimized Leak-Free 3D Printed Fluidics
Source: ACS Appl Polym Mater. 2025 Oct 24;7(21):14130–7. doi: 10.1021/acsapm.5c02274 (PMC12624528; doi:10.1021/acsapm.5c02274)
Supplement: Supplementary file 1 [file ap5c02274_si_001.pdf]

Supporting information for

Microscale computed tomography (μCT) imaging of leak pathways for optimised leak-free 3D printed fluidics

Rowan Leeder,[a] Kathryn E. Rankin,[b] Adrian M. Nightingale[a*]

[a] Mechanical Engineering, Faculty of Engineering and Physical Sciences, University of Southampton, SO17 1BJ, UK
[b] μ-VIS X-ray Imaging Centre, Faculty of Engineering and Physical Sciences, University of Southampton, SO17 1BJ, UK

*a.nightingale@southampton.ac.uk

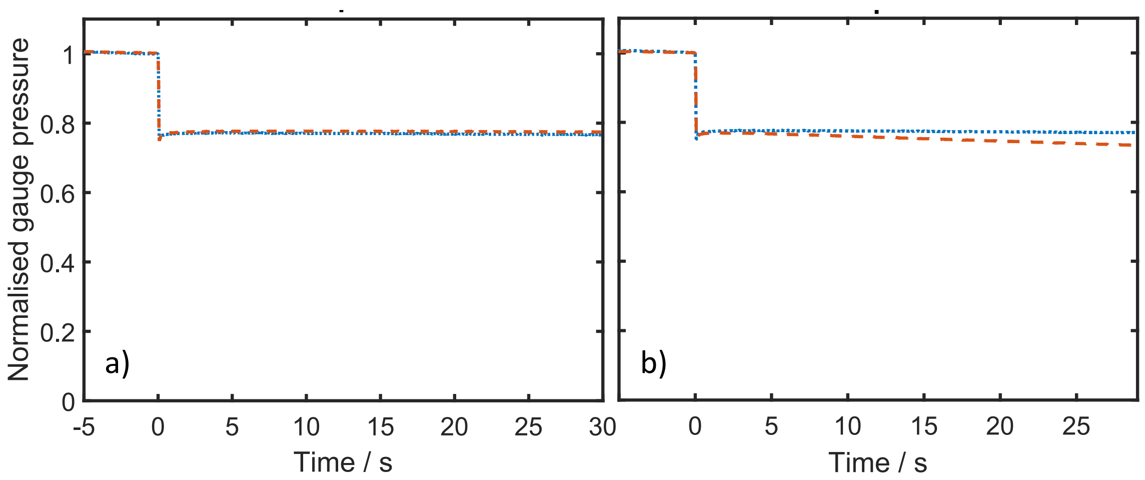

Figure S1: Comparison of quantitative leak tests performed on two separate test-pieces (a & b), each printed using identical print parameters with 0.06 mm layer height,100 % extrusion rate, and 100 % infill. For each test piece two separate measurements are shown (red dashed and blue dotted lines). Data in a) is the same as that shown in the main manuscript (Fig. 2b.i) and is reproduced here so it can be easily compared with the repeat (b).

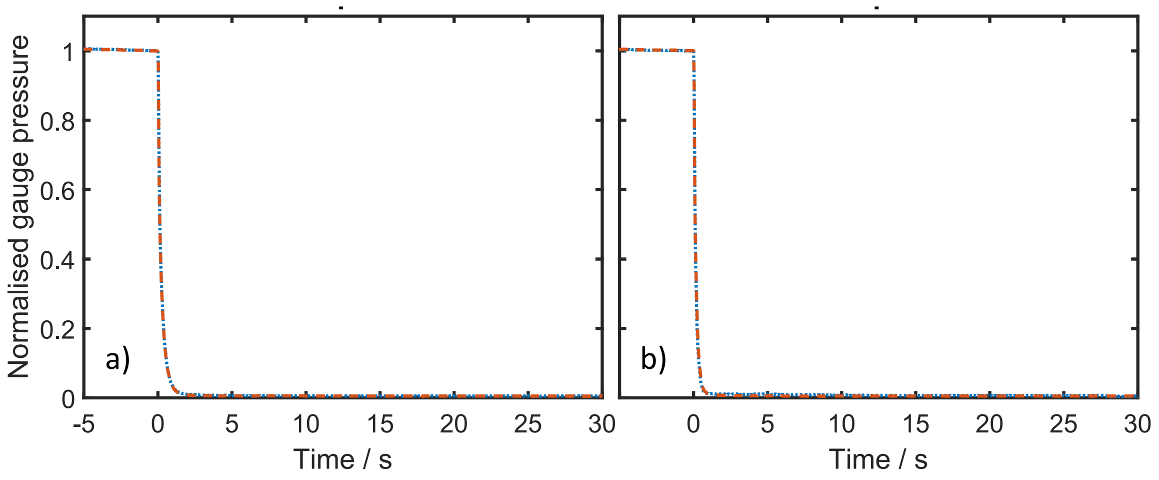

Figure S2: Comparison of quantitative leak tests performed on two separate test-pieces (a & b), each printed using identical print parameters with 0.2 mm layer height,100 % extrusion rate, and 100 % infill. For each test piece two separate measurements are shown (red dashed and blue dotted lines). Data in a) is the same as that shown in the main manuscript (Fig. 2b.ii) and is reproduced here so it can be easily compared with the repeat (b).

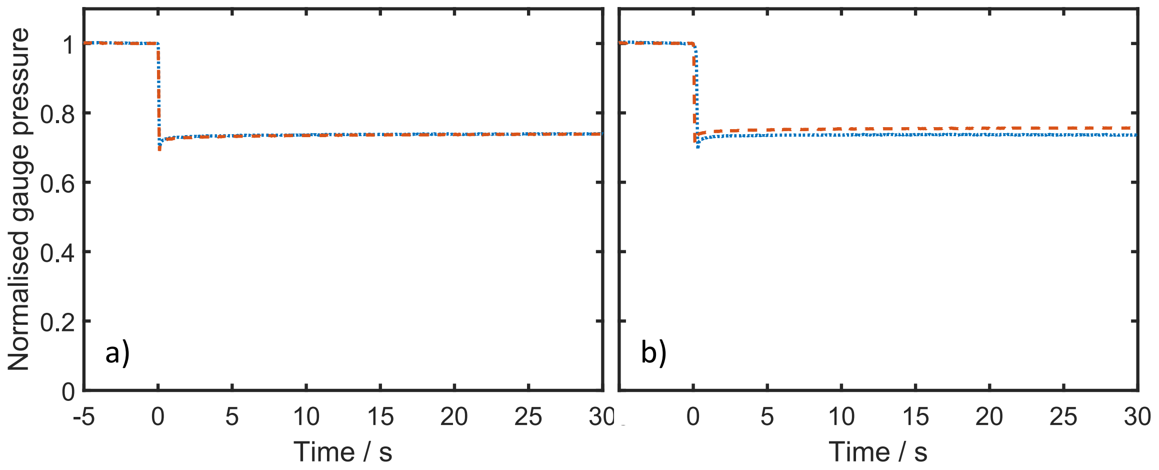

Figure S3: Comparison of quantitative leak tests performed on two separate test-pieces (a & b), each printed using identical print parameters with 0.06 mm layer height,110 % extrusion rate, and 20 % infill. For each test piece two separate measurements are shown (red dashed and blue dotted lines). Data in a) is the same as that shown in the main manuscript (Fig. 7c) and is reproduced here so it can be easily compared with the repeat (b).
